# Corrigendum to “Development and Evaluation of an Immuno-Capture Enzyme-Linked Immunosorbent Assay to Quantify the *Mycoplasma capricolum* subsp. Capripneumoniae (Mccp) Protein in Contagious Caprine Pleuropneumonia (CCPP) Vaccine”

**DOI:** 10.1155/2021/9813273

**Published:** 2021-03-23

**Authors:** Jean de Dieu Baziki, Charles Bodjo S., Nick Nwankpa, Naomi Maina, Ethel Chitsungo, Cisse Rahamatou Moustapha Boukary, Takele A. Tefera, Rume Veronica Nwankpa, Nduta Mwangi

**Affiliations:** ^1^African Union-Pan African Veterinary Vaccine Centre (AU-PANVAC), P.O. Box: 1746, Debre Zeit, Ethiopia; ^2^Pan African University Institute for Basic Sciences, Technology and Innovation (PAUSTI), JKUAT Main Campus, P.O. Box: 62000-00200, Nairobi, Kenya; ^3^Jomo Kenyatta University of Agriculture and Technology (JKUAT), P.O. Box: 62000-00200, Nairobi, Kenya; ^4^National Veterinary Institute, P.O. Box: 19, Debrezeit, Ethiopia; ^5^Addis Ababa University, Department of Microbial Cellular and Molecular Biology, P.O. Box: 1176, Addis Ababa, Ethiopia; ^6^Kenya Veterinary Vaccines Production Institute (KEVEVAPI), P.O. Box: 53260-00200, Nairobi, Kenya

In the article titled “Development and Evaluation of an Immuno-Capture Enzyme-Linked Immunosorbent Assay to Quantify the *Mycoplasma capricolum* subsp. Capripneumoniae (Mccp) Protein in Contagious Caprine Pleuropneumonia (CCPP) Vaccine” [1], information was omitted in the Acknowledgments section. The corrected section appears as follows:
